# Musical ability and emotion recognition in speech prosody: The role of pitch discrimination

**DOI:** 10.3758/s13423-026-02865-z

**Published:** 2026-02-27

**Authors:** Aíssa M. Baldé, E. Glenn Schellenberg, César F. Lima

**Affiliations:** 1 Centro de Investigação e Intervenção Social (CIS-IUL), Instituto Universitário de Lisboa (ISCTE-IUL), Av.ª das Forças Armadas, 1649-026 Lisbon, Portugal; 2 Department of Psychology, University of Toronto Mississauga, Mississauga, Canada

**Keywords:** Emotion recognition, Music training, Musical aptitude, Psychoacoustics, Pitch discrimination

## Abstract

Why does musical expertise predict enhanced emotion recognition in speech prosody? Evidence for a causal role of music training is weak, and correlations with musical aptitude could reflect basic auditory abilities rather than musicality per se. Here, we tested whether individual differences in basic auditory discrimination account for the music–prosody association. A total of 164 adults completed forced-choice judgments of emotions in prosody and facial expressions, self-reports of musical experience, objective tests of musical ability (melody and rhythm perception), and adaptive psychoacoustic tasks that estimated discrimination thresholds for pitch, duration, loudness, timbre, and backward masking. Both music training and musical ability correlated with better recognition of prosodic but not facial emotions. The training effect was weak, however, and disappeared after controlling for confounding variables, including general cognitive ability. By contrast, musical ability, specifically melody perception, remained associated with prosodic emotion recognition after accounting for training and other covariates. Crucially, psychoacoustic thresholds correlated with both prosody recognition and melody perception. When considered simultaneously, pitch discrimination alone independently predicted prosodic emotion recognition, but melody perception did not. These findings suggest that music training is an artifactual correlate of prosodic emotion recognition, and that basic pitch sensitivity underlies the link between musical ability and emotional prosody.

**Supplementary Information:**

The online version contains supplementary material available at 10.3758/s13423-026-02865-z.

Recognizing emotions from speech prosody, through intonation, timing, loudness, and timbre, is essential for communication and socioemotional functioning (Carton et al., 1999; Leipold et al., 2022; Neves et al., 2021; Paz et al., 2025; Terracciano et al., 2003). Listeners reliably identify prosodic emotions from early in development (Sauter et al., 2013) and across cultures (Cowen et al., 2019; Scherer et al., 2001). Emotions in music show a remarkably similar profile. They are recognized from early childhood (Vidas et al., 2018), cross-culturally (Fritz et al., 2009; Singh & Mehr, 2023), and through acoustic cues that largely overlap with those used in prosody (Juslin & Laukka, 2003). Sadness, for example, is typically conveyed by slower tempo or speech rate, lower sound level or vocal intensity, lower pitch, and reduced high-frequency energy. These parallels raise the possibility that sensitivity to emotional cues generalizes across domains.

A growing literature reports positive correlations between music training and emotion recognition, both in music (Bhatara et al., 2011; Castro & Lima, 2014) and in prosody (Correia et al., 2022a, 2022b; Jansen et al., 2023; Lima & Castro, 2011; Martins et al., 2021; Nussbaum et al., 2024). Although associations with prosody are typically small and not always replicable (Martins et al., 2021; Nussbaum et al., 2025), they are theoretically important as potential evidence of transfer from music training to nonmusical communication. The OPERA hypothesis (Patel, 2011, 2014) formalizes one plausible mechanism: Because music and speech share sensory and cognitive processes, music training could enhance speech processing by involving greater sensory precision, extensive practice, focused attention, and emotional engagement.

Nevertheless, causal evidence for training effects is limited. Most studies are correlational, comparing trained and untrained individuals, which leaves open roles for selection effects and preexisting differences (Martins et al., 2021; Schellenberg, 2020; Schellenberg & Lima, 2024). Because music training is associated with socioeconomic status, general cognitive ability, and personality (e.g., Corrigall et al., 2013), any of these factors could explain observed advantages. Indeed, a recent review questioned whether music training improves nonmusical abilities, including emotion recognition (Schellenberg & Lima, 2024), and a large multi-lab study failed to replicate previously reported links with neural responses to speech (Whiteford et al., 2025).

An alternative account shifts the focus from training to musical aptitude or ability. Perceptual measures of musical ability often outperform years of lessons in predicting vocal-emotion recognition. For example, a randomized intervention found no improvement in children’s emotion recognition after two years of music lessons, yet baseline musical ability predicted emotion recognition independently of training (Neves et al., 2025). Similarly, among adults, musical ability correlates with vocal-emotion recognition independently of training, whereas apparent training effects disappear after accounting for ability (Correia et al., 2022a, 2022b; Nussbaum et al., 2024, 2025). Meta-analytic evidence confirms that prosody perception, both emotional and linguistic, is more strongly associated with musical ability than with music training (Jansen et al., 2023).

Musical ability is commonly assessed with same-different judgments of short musical sequences that vary in melody, rhythm, or other features (e.g., Musical Ear Test [MET]; Wallentin et al., 2010; Profile of Music Perception Skills [PROMS]; Law & Zentner, 2012). Although typical sequences are structured musical patterns, music perception begins with encoding elementary acoustic features, such as pitch and duration, before music-specific operations occur (Koelsch, 2011; Peretz & Coltheart, 2003). Because stimuli typically comprise multiple tones, musical-ability tests also recruit short-term and working memory, which help to explain why musical-ability scores correlate with general cognitive performance (Baldé et al., 2025; Correia et al., 2022a, 2022b, 2023; Grassi et al., 2025; Swaminathan & Schellenberg, 2020).

In short, associations between musical ability and prosodic emotion recognition may reflect low-level auditory sensitivity or general cognitive mechanisms rather than “musicality” per se. While the speech and language literature routinely separates sensory encoding from higher-order linguistic processing (e.g., Saito, 2023), the music literature often treats psychoacoustic discrimination and complex music-perception tests interchangeably (Correia et al., 2022a, 2022b; Law & Zentner, 2012; Martin et al., 2018; Thompson et al., 2012). According to mechanistic models of music perception and cognition (Koelsch, 2011; Peretz & Coltheart, 2003), domain-general auditory processes should be distinguished from music-specific operations.

Basic auditory processing is best measured with adaptive psychoacoustic tasks using simple, nonmusical stimuli (e.g., pure tones) to estimate sensory thresholds for isolated dimensions such as pitch, duration, intensity, and timbre (Baldé et al., 2025; Soranzo & Grassi, 2014). Lower (better) thresholds are typically evident in musicians across dimensions (Bianchi et al., 2016; Endo et al., 2021; Lee & Müllensiefen, 2020; Rammsayer & Altenmüller, 2006), whereas higher thresholds are evident among individuals with congenital amusia, a disorder characterized by impaired pitch discrimination (Hyde & Peretz, 2004). Moreover, psychoacoustic thresholds predict musical ability (melody and rhythm perception) even after controlling for training, cognitive abilities, personality, and demographics (Baldé et al., 2025).

Importantly, psychoacoustic thresholds—particularly pitch discrimination—predict prosodic emotion recognition in typically developing adults (Globerson et al., 2013) and in individuals with amusia (Lima et al., 2016; Lolli et al., 2015; Pralus et al., 2019). In a reanalysis of Correia et al., (2022a, 2022b; see the Supplementary Materials), psychoacoustic thresholds, but not a beat-alignment musical test, predicted vocal-emotion recognition independently of training. When considered simultaneously, only pitch discrimination accounted for unique variance in emotion recognition. Voice-morphing studies highlight further that when prosodic emotions are expressed exclusively through pitch, recognition remains high and correlated with musical expertise (Nussbaum et al., 2024).

Motivated by these findings, we asked whether musical expertise (defined as both training and ability) predicts emotion recognition from prosody, and whether any association is music-specific or explained by basic auditory sensitivity. We tested 164 adults with diverse musical backgrounds. Forced-choice tasks measured emotion recognition from prosody and, as a nonauditory control, facial expressions. Predictors included self-reported music training and abilities (Goldsmiths Musical Sophistication Index [Gold-MSI]; Müllensiefen et al., 2014), objective melody and rhythm perception (MET subtests; Wallentin et al., 2010), and psychoacoustic thresholds for pitch, duration, loudness, timbre, and backward masking. We also measured cognitive abilities, personality, and sociodemographic variables, which can covary with musical expertise. For example, training duration correlates with higher general cognitive ability and openness-to-experience (e.g., Corrigall et al., 2013), whereas musical ability improves with age, education, and general cognitive ability (e.g., Correia et al., 2022a, 2022b).

We hypothesized that (1) musical expertise—particularly musical ability—would correlate with prosodic emotion recognition, (2) basic auditory processing would correlate with both musical ability and prosody perception, and (3) pitch discrimination would account for the association between musical ability and prosodic emotion recognition, such that musical ability would not explain unique variance after accounting for pitch thresholds.

## Method

### Participants

The initial sample comprised 167 participants but three were excluded due to missing data (*n* = 1) or hearing difficulties (*n* = 2). The final sample of 164 participants (138 women, 26 men) ranged in age from 18 to 65 years (*M* = 22.7, *SD* = 7.28). Age was square-root transformed for statistical analysis. Most participants were undergraduate students. Education was coded as a three-level ordinal variable: no bachelor’s degree (*n* = 123), bachelor’s degree (*n* = 26), or master’s degree (*n* = 15). All participants were native speakers of European Portuguese and reported normal hearing.

Participants varied widely in formal music training. Sixty-four reported no lessons, six reported half a year, nine reported 1 year, 17 reported 2 years, and 18 reported between 3 and 5 years. Fifty participants had 6 or more years of training, including 26 with at least 10 years.

Sample size was similar to that of Baldé et al. (2025) and Correia et al., (2022a, 2022b). Post hoc power analysis conducted with G*Power (Faul et al., 2009) confirmed that we had 90.1% power to detect medium-sized partial correlations (*r* = .25, seven covariates, two-tailed, alpha = .05).

### Materials

#### Emotion recognition

Two forced-choice tasks required participants to categorize emotions conveyed by prosodic cues or facial expressions. Both tasks had 84 trials. The stimuli were taken from validated corpora (prosody, Castro & Lima, 2010; faces, Karolinska Directed Emotional Faces database, Lundqvist et al., 1998) and expressed one of seven emotions: anger, disgust, fear, happiness, surprise, sadness, and neutrality, with 12 stimuli per emotion. Prosodic stimuli were semantically neutral short sentences (*M* = 1511 ms, *SD* = 305; e.g., “Ele chega amanhã,” *He arrives tomorrow*) produced by two female and two male speakers (three different sentences per speaker per emotion). Peak amplitude was normalized to 75 dB using Praat (Boersma & Weenink, 2025). Facial stimuli were color photographs of male and female adults without beards, moustaches, earrings, eyeglasses, or visible makeup.

The procedure was identical for both tasks. After each stimulus was presented, participants selected the label that matched the expressed emotion from seven response options. Both tasks began with four practice trials. Prosodic stimuli were presented once; facial stimuli remained visible for 2 s. No feedback was provided. The order of the stimuli and response options were randomized across participants. Each trial consisted of a fixation cross (500 ms), followed by the stimulus and then the response. The two tasks took approximately 12 min to complete.

#### Psychoacoustic tasks

Following Baldé et al. (2025), we administered nine psychoacoustic tasks from the PSYCHOACOUSTICS toolbox (Soranzo & Grassi, 2014). Three measured pitch perception using pure tones (pitch), tone sequences (contour speed), or musical scales (scale mistuning). Four measured temporal processing, including duration discrimination with pure tones (duration) and rhythmic sequences (complex duration), gap detection with broadband noise (gap detection), and temporal resolution with pure tones and noise (backward masking). The remaining two tasks measured loudness (intensity) and timbre perception (timbre). Sounds were presented at 75 dB SPL through headphones (Mars Gaming MH4X) and included 10-ms onset and offset ramps.

All but one task (scale mistuning) used a three-alternative forced-choice format. On each trial, three stimuli were presented successively: two identical standards and a comparison that differed on a single acoustic dimension. The comparison could be presented in the first, second, or third position. Listeners’ task was to indicate the odd stimulus (1, 2, or 3), with target position randomized across trials. The difference between standards and comparison was adjusted adaptively using a two-down, one-up staircase procedure, targeting the minimum change required for 70.7% accuracy (Levitt, 1971; Soranzo & Grassi, 2014). Changes were salient at first to ensure task comprehension. After two consecutive correct responses, the difference between standards and comparison decreased by a factor of 2. After one incorrect response, it increased by a factor of 2. After four reversals, step size was reduced to √2 to fine tune the estimate. Tasks ended after 12 reversals, with the threshold calculated as the geometric mean of the difference between standard and comparison stimuli on the final eight reversals. Lower thresholds indicated better performance, such that we expected negative associations between thresholds and other measures.

On each trial of the Pitch task, listeners heard three 250-ms pure tones and identified the one with the highest pitch. Standards were fixed at 1000 Hz and the comparison was higher. For contour speed, listeners heard three sequences of four tones and identified the one that had a contour change, which reversed the order of the second and third tones (550–710 Hz or 710–550 Hz). The duration of the middle two tones decreased adaptively with correct responding, such that the test measured the shortest duration that still allowed for correct identification of a salient difference in pitch, as in tests of auditory inspection time (e.g., Deary, 1995). Scale mistuning had a yes/no response format that differed from the other tasks. Each trial presented a single ascending equal-tempered C-major scale (500-ms complexes of five harmonics). Listeners judged whether the scale was in or out of tune. Sol (G, the 5th scale degree) was mistuned upward to varying degrees.

Trials in the duration task comprised three pure tones. The two standards were 250 ms and the comparison was longer. For complex duration, trials contained three sequences of six 20-ms, 1000-Hz tone pulses arranged as three pairs. In the standards, silences were 40 ms within pairs and 120 ms between pairs. The comparison was identical in total duration, but within-pair silences were longer and between-pair silences were shorter. The magnitude of these differences decreased adaptively. For gap detection*,* listeners heard three 750-ms bands of Gaussian noise and identified the one containing a silent gap, which became briefer over time. For backward masking, each trial contained three 300-ms bands of bandpass noise and listeners identified which one had a 20-ms target tone that immediately preceded the noise. The target became quieter with correct responding.

Each trial in the Intensity task had three 1000 Hz pure tones (250 ms) and listeners identified the loudest. Finally, for Timbre, listeners heard three complex tones comprising five harmonics (e.g. 200, 400, 800, 1600, and 3200 Hz). Harmonics had equal amplitudes in standards, but the third harmonic was louder in the comparison.

#### Self-reported music training and abilities

We assessed music training using the Music Training subscale from the Gold-MSI, a 38-item questionnaire that indexes different facets of musical expertise and behavior (Müllensiefen et al., 2014; Portuguese translation: Lima et al., 2020). Gold-MSI items are rated on 7-point scales and grouped into five subscales: Active Engagement (nine items, e.g., *I’m intrigued by musical styles I’m not familiar with and want to find out more*); Perceptual Abilities (nine items, e.g., *I can tell when people sing or play out of tune*), Music Training (seven items, e.g., *I had [0–10 or more] years of formal training on a musical instrument [including voice] during my lifetime*), Singing Abilities (seven items, e.g., *I only need to hear a new tune once and I can usually sing it back hours later*), and Emotions (six items, e.g., *Music can evoke my memories of past people and places*). In addition to an item asking about years of formal instruction, the Music Training subscale has additional items that ask about practice duration and frequency, compliments on musical performances, and self-identification as a musician. We opted to use subscale scores in the analyses after confirming that they correlated highly with training duration (from the subscale’s own training item; 0 = *no training*, 7 = *10 years or more*), *r* = .863, *p* < .001, and with responses to an additional question that asked about exact years of training (square-root transformed), *r* = .889, *p* < .001.

We also standardized and averaged the two subscales from the Gold-MSI that focused on musical abilities (Perceptual Abilities and Singing Abilities). The composite score is hereafter referred to as *Self-Reported Musicality*. The remaining two subscales (Active Engagement and Emotions) were irrelevant to our hypotheses and not considered further.^1^

#### Objective musical abilities

We measured melody and rhythm perception objectively with the online version (Correia et al., 2022a, 2022b) of the MET (Wallentin et al., 2010). Because participants completed the test individually in the laboratory, testing closely matched the original computerized version. The test’s first and second halves provided scores for the Melody and Rhythm subtests, respectively. Each subtest had 52 trials preceded by two practice trials. On each trial, listeners heard two short musical excerpts played on piano (Melody) or woodblock (Rhythm). Their task was to determine whether the first and second were identical (yes/no). In half of the trials, the second excerpt differed by changing the pitch of one or more tones in the Melody subtest, and one or more inter-onset intervals in the Rhythm subtest. No feedback was provided during test trials. Subtest scores were the number of correct responses. The entire MET took approximately 20 min to complete. Average performance accuracy (*M* = 69.1%) was similar to published norms (*M* = 69.7%; Swaminathan et al., 2021).

#### Control measures

In addition to demographic variables (gender, age, and education; see Participants), we assessed general cognitive ability and personality.

##### General cognitive abilities

Nonverbal (abstract) reasoning was measured with the Matrix Reasoning Item Bank (MaRs-IB; Chierchia et al., 2019), an online 8-min test modeled after Raven’s Advanced Progressive Matrices (Raven, 1965). On each trial, participants had up to 30 s to respond, and scores were calculated as the proportion correct (number correct/trials attempted). Responses faster than 250 ms were excluded, and proportions were logit-transformed for statistical analysis.

Short-term and working memory were assessed with the forward and backward portions, respectively, of the Digit Span subtest from the Wechsler Adult Intelligence Scale–Third Edition (WAIS-III; Wechsler, 2008). In the forward portion, participants repeated aloud lists of numbers, of increasing length, in the presented order. In the backward portion, they recalled the digits in reverse order. Span length increased by one digit after every two trials. Testing stopped after failure on both trials of a given span. Scores on each portion were the maximum number of digits recalled correctly.

##### Personality

The online version (Correia et al., 2022a, 2022b) of the Big Five Inventory (BFI; John et al., 2008; Portuguese translation: Brito Costa et al., 2016) evaluated the five major dimensions of personality (McCrae & John, 1992): Extraversion, Agreeableness, Concientiousness, Neuroticism, and Openness-to-Experience (hereafter, Openness). Participants rated 44 items on a 5-point scale (1 = *disagree strongly* to 5 = *agree strongly*). Each item represented a characteristic of one of the five dimensions (e.g., Openness: *I see myself as someone who values artistic, aesthetic experience*).

### Procedure

Participants were tested individually in a quiet room, with sessions lasting approximately 2 h. After obtaining informed consent, participants were interviewed briefly about their musical background. The tasks were then administered in the following order: first block of psychoacoustic tests (Pitch, Intensity, Duration, Complex Duration, Contour Speed), Digit Span, second block of psychoacoustic tests (Backward Masking, Gap Detection, Timbre, Scale Mistuning), Gold-MSI, BFI, MaRs-IB, MET, and finally the emotion-recognition tasks. The psychoacoustic tests were presented using MATLAB (r2022b, Version 9.13.0), with the order of tests randomized within each block for each participant. Subsequent questionnaires and tasks were implemented using the online platform Gorilla Experiment Builder (Anwyl-Irvine et al., 2020). Participants were allowed short breaks between tasks and received feedback about their musical abilities and personality at the end of the session.

### Data preparation and analysis

All analysis were conducted in JASP (Version 0.19.3; JASP Team, 2025). We report both standard frequentist (Null Hypothesis Significance Testing [NHST]) and Bayesian statistics (default priors). For each analysis, we report a Bayes factor (BF_10_) with three-digit accuracy alongside *p* values. Whereas *p* values indicate the probability of the observed data under the null hypothesis, Bayes factors quantify the strength of evidence in favor of the alternative hypothesis. BF_10_ values > 1.00 favor the alternative hypothesis. By convention (Jarosz & Wiley, 2014; Jeffreys, 1998), BF_10_ ≤ 3.00 indicates weak or anecdotal evidence; BF_10_ > 3.00 indicates substantial evidence; BF_10_ > 10.0, strong evidence; BF_10_ > 30.0, very strong evidence; and BF_10_ > 100, decisive evidence. Conversely, BF_10_ values < 1.00 favor the null hypothesis, with BF_10_ < .333, .100, .033, .010 corresponding, respectively, to substantial, strong, very strong, or decisive evidence for the null. We considered the results to provide reliable evidence for the alternative hypothesis only when *p* < .05 and BF_10_ > 3.00, or for the null hypothesis when* p* > .05 and BF_10_ < .333.

Because psychoacoustic thresholds typically follow a logarithmic distribution (Moore, 2003), they were log-transformed for statistical analyses (e.g., Baldé et al., 2025; Globerson et al., 2013; Micheyl et al., 2006). Thresholds and musical-ability scores deviating more than 3 standard deviations from the group mean (i.e., unusually high thresholds or unusually low musical-ability scores) were considered outliers and excluded. Missing data (MET Melody *n* = 1, Pitch *n* = 4, Intensity *n* = 2, Duration *n* = 3, Complex Duration *n* = 2, Contour Speed *n* = 9, Gap Detection *n* = 4, Timbre *n* = 8, Scale Mistuning *n* = 5) meant that sample size varied across analyses depending on which variables were included.

Analyses of emotion recognition used average scores because we had no hypotheses about specific emotions, and because the literature typically reports a general advantage for musicians (e.g., Correia et al., 2022a, 2022b; Lima & Castro, 2011; Martins et al., 2021; Nussbaum & Schweinberger, 2021). Results for individual categories are nevertheless provided in Supplementary Materials.

## Results

Descriptive statistics are presented in Table 1. To identify relevant covariates, we examined correlations between demographic, cognitive, and personality factors and our primary measures of interest (emotion recognition, psychoacoustic thresholds, music training, musical abilities; Supplementary Tables S1–3). Age, gender, and education (demographics); nonverbal reasoning, digit span forward, and digit span backwards (cognition); and openness (personality) each correlated significantly with at least one primary measure. These seven factors were treated as *control variables* in all covariate-adjusted analyses.

**Table 1:** Descriptive statistics

	*M*	*SD*
Demographics		
Age	22.7	7.28
Education (3 levels)	1.34	0.64
Emotion Recognition		
Prosody (%)	72.26	8.90
Faces (%)	83.16	5.92
MET		
Melody (%)	68.48	13.37
Rhythm (%)	69.79	11.01
Gold-MSI		
Music Training	3.30	1.82
Self-Reported Musicality	0.00	0.92
Psychoacoustic Thresholds		
Pitch (Hz)	23.91	32.18
Intensity (dB)	1.96	1.00
Duration (ms)	35.66	16.80
Complex Duration (ms)	53.24	54.88
Contour Speed (ms)	117.49	101.15
Backward Masking (dB)	− 46.00	17.43
Gap Detection (ms)	2.33	0.64
Timbre (dB)	5.73	2.74
Scale Mistuning (semitones)	1.04	1.18
Cognitive abilities		
MaRs-IB–Nonverbal Reasoning (%)	64.70	14.28
Digit Span Forward	5.98	1.09
Digit Span Backwards	4.65	1.06
Personality		
Extraversion	3.17	0.82
Agreeableness	3.82	0.51
Conscientiousness	3.43	0.68
Neuroticism	3.26	0.78
Openness	3.78	0.63

The main analyses tested whether emotion recognition was associated with (1) music training, (2) musical abilities, and (3) basic auditory skills (psychoacoustic thresholds). We first examined each predictor separately using partial correlations adjusted for the control variables. We then implemented a joint model testing whether musical abilities remained predictive of prosodic emotion recognition when basic auditory skills were considered simultaneously. Secondary analyses compared highly trained (≥ 6 years of lessons) and untrained (≤ 2 years) participants to confirm that the correlational findings generalized to the analytical approach used commonly in the literature.

### Music training

Table 2 reports partial correlations between music training (Gold-MSI subscale) and other primary measures after adjusting for control variables. As expected, music training correlated with better objective musical ability, higher self-reported musicality, and superior thresholds on pitch-based psychoacoustic tasks (Pitch, Contour Speed, Scale Mistuning) and Backward Masking.

**Table 2:** Partial pairwise correlations between music training (Music Training Subscale, Gold-MSI) and emotion recognition, musical ability, and psychoacoustic thresholds

Variable	*r*	*p*	BF_10_
Emotion Recognition			
Prosody	.031	.702	.289
Faces	− .030	.707	.288
Musical Ability			
Melody	**.520**	** < .001**	** > 100**
Rhythm	**.364**	** < .001**	** > 100**
Self-Reported Musicality	**.571**	** < .001**	** > 100**
Psychoacoustic Thresholds			
Pitch	** − .452**	** < .001**	** > 100**
Intensity	− .102	.207	.570
Duration	− .103	.206	.574
Complex Duration	− .041	.611	.306
Contour Speed	** − .312**	** < .001**	** > 100**
Backward Masking	** −.313**	** < .001**	** > 100**
Gap Detection	− .110	.175	.632
Timbre	− .072	.383	.394
Scale Mistuning	** − .310**	** < .001**	** > 100**

Gender, age, education, nonverbal reasoning, digit span forward, digit span backwards, and openness were held constant. Significant findings are in bold font

Before covariate adjustment, music training correlated with prosodic emotion recognition, *r* = .194, *p* = .013, BF_10_ = 2.11, although Bayesian evidence was weak. For facial emotion recognition, there was substantial evidence for no association, *r* = .061, *p* = .441, BF_10_ = .131. After adjusting for control variables, the training-prosody association disappeared, and Bayesian evidence supported the null hypothesis (Table 2).

The same pattern emerged when highly trained (*n* = 50) and untrained (*n* = 96) groups were compared. For prosody, trained participants (*M* = 75.91% correct, *SD* = 8.20) outperformed untrained ones (*M* = 70.99, *SD* = 8.53) before covariate adjustment, *t*(144) = 2.29, *p* = .024, BF_10_ = 1.96, but Bayesian evidence was again weak. For faces, trained (*M* = 84.50, *SD* = 6.31) and untrained (*M* = 82.80, *SD* = 5.62) groups did not differ, *t*(144) = 1.35, *p* = .180, BF_10_ = .425. After covariate adjustment, an analysis of covariance (ANCOVA) confirmed that evidence for a null effect was close to substantial for prosody, *F* < 1, *p* = .593, BF_10_ = .336, and substantial for faces, *F* < 1, *p* = .471, BF_10_ = .294. Thus, across correlational and group-based analyses, associations between music training and prosodic emotion recognition were weak and appeared to stem from individual differences in control variables.

### Musical abilities

**Fig. 1:**
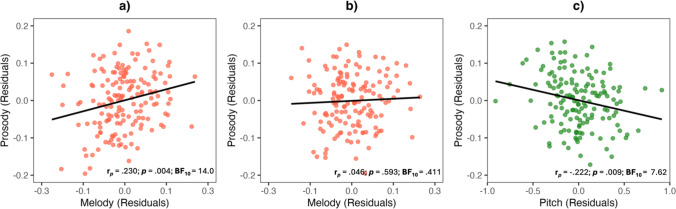
Associations between prosodic emotion recognition and melody perception **a)** before and **b)** after adjusting for psychoacoustic thresholds (pitch, duration, intensity, gap detection). **c)** Association between prosodic emotion recognition and pitch perception after adjusting for melody perception and other psychoacoustic thresholds (duration, intensity, gap detection). *Note.* In all panels, prosody, melody, and pitch were additionally adjusted for age, gender, education, music training, nonverbal reasoning, digit span forward, digit span backwards, and openness

Melody perception showed a substantial association with prosodic emotion recognition even after covariate adjustment, *r* = .211, *p* = .008, BF_10_ = 7.53, but not with facial emotion recognition, *r* = .076, *p* = .345, BF_10_ = .551. This melody-prosody association remained robust after controlling additionally for music training, *r* = .230, *p* = .004, BF₁₀ = 14.0 (Fig. 1a), confirming that it did not stem from greater training among higher performers. Rhythm perception, by contrast, did not correlate with prosody, *r* = .112, *p* = .161, BF_10_ = .816, or facial emotion recognition, *r* = .123, *p* = .124, BF_10_ = 1.04. Similarly, self-reported musicality showed no clear association with prosody, *r* = .141, *p* = .077, BF_10_ = 1.36, or faces, *r* =  − .007, *p* = .930, BF_10_ = .375.

In short, objective musical ability, particularly melody perception, predicted prosodic emotion recognition independently of control variables and music training.

### Basic auditory skills

Table 3 presents partial correlations among auditory thresholds, musical abilities, and emotion recognition after accounting for control variables. Lower (better) thresholds across all nine psychoacoustic tasks predicted improved melody perception, with decisive evidence (BF₁₀ > 100) for pitch-related tasks (Pitch, Contour Speed, Scale Mistuning), Backward Masking, and Timbre. Both rhythm and self-reported musicality were associated with seven psychoacoustic tasks. After adjusting additionally for music training, all associations between auditory thresholds and melody perception remained significant (Supplementary Table S5). Rhythm perception remained associated with only the Pitch and three temporal tasks (Duration, Complex Duration, and Backward Masking), whereas self-reported musicality was associated with six thresholds.

**Table 3:** Partial pairwise correlations between psychoacoustic thresholds, emotion recognition, and musical ability

		Prosody	Faces	Melody	Rhythm	Self-Reported Musicality
Pitch	*r*	** − .339**	− .158	** − .568**	** − .327**	** − .439**
	*p*	** < .001**	.051	** < .001**	** < .001**	** < .001**
	BF_10_	** > 100**	1.92	** > 100**	** > 100**	** > 100**
Intensity	*r*	** − .229**	** − .205**	** − .253**	** − .193**	** − .281**
	*p*	**.004**	**.010**	**.002**	**.016**	** < .001**
	BF_10_	**13.2**	**6.40**	**29.4**	**4.17**	**88.8**
Duration	*r*	** − .279**	** − .178**	** − .245**	** − .275**	− .131
	*p*	** < .001**	**.028**	**.002**	** < .001**	.105
	BF_10_	**79.8**	**3.06**	**21.1**	**69.2**	1.19
Complex Duration	*r*	− .158	− .067	** − .274**	** − .217**	− .124
	*p*	.049	.408	** < .001**	**.007**	.125
	BF_10_	1.90	.504	**67.0**	**8.38**	1.08
Contour Speed	*r*	− .073	− .086	** − .469**	** − .276**	** − .274**
	*p*	.381	.301	** < .001**	** < .001**	** < .001**
	BF_10_	.511	.607	** > 100**	**59.2**	**52.8**
Backward Masking	*r*	− .148	.101	** − .407**	** − .317**	** − .339**
	*p*	.065	.207	** < .001**	** < .001**	** < .001**
	BF_10_	1.55	.743	** > 100**	** > 100**	** > 100**
Gap Detection	*r*	** − .240**	.016	** − .271**	− .045	** − .247**
	*p*	**.003**	.844	** < .001**	.583	**.002**
	BF_10_	**18.3**	.384	**54.6**	.332	**23.3**
Timbre	*r*	− .149	− .068	** − .315**	− .145	** − .237**
	*p*	070	.410	** < .001**	.078	**.004**
	BF_10_	1.51	.519	** > 100**	1.24	**14.6**
Scale Mistuning	*r*	− .130	.045	** − .462**	** − .225**	** − .378**
	*p*	.109	.582	** < .001**	**.005**	** < .001**
	BF_10_	1.08	.422	** > 100**	**10.2**	** > 100**

Gender, age, education, nonverbal reasoning, digit span forward, digit span backwards, and openness were held constant. Significant findings are in bold font

Four psychoacoustic thresholds predicted prosodic emotion recognition after covariate adjustment: Pitch (decisive evidence), Duration (very strong evidence), and Intensity and Gap Detection (strong evidence; Table 3). Correlations with facial emotion recognition were weaker, with substantial evidence only for Intensity and Duration. All associations remained significant after controlling additionally for music training (Supplementary Table S5).

Thus, auditory thresholds correlated with both musical abilities and prosodic emotion recognition, independently of control variables and music training.

### Joint prediction of emotional prosody from melody and basic auditory skills

Finally, we tested whether melody perception continued to predict prosodic emotion recognition when the psychoacoustic thresholds associated with prosody were considered simultaneously. A hierarchical multiple regression predicted prosody scores from control variables and music training at Step 1, adding at Step 2 melody and the four psychoacoustic thresholds (Pitch, Duration, Intensity, Gap Detection). Results are presented in Table 4. Step 1 explained 20.0% of the variance, *R* = .447, *F*(8, 142) = 4.44, *p* < .001. Significant predictors were higher openness, younger age, and female gender. On Step 2, explained variance increased by 14.3%, *F*(5, 137) = 5.97, *p* < .001. Pitch discrimination emerged as the strongest independent predictor of prosodic emotion recognition. Melody perception no longer contributed unique variance, with Bayesian evidence slightly favoring the null hypothesis (Table 4; Fig. 1 b, c). Thus, when considered jointly, basic pitch sensitivity, but not melody perception, explained individual differences in prosodic emotion recognition.

**Table 4:** Summary of hierarchical multiple regression model predicting prosodic emotion recognition

	β	*p*	BF_10_	*r*_*p*_	*R*^2^	Adjusted *R*^2^
*Step 1*					.200	.155
Age	** − .236**	**.019**	**4.11**	** − .196**		
Gender	**.175**	**.024**	**3.37**	**.188**		
Education	.045	.677	.384	.035		
Nonverbal Reasoning	.189	.041	2.27	.171		
Digit Span Forward	.103	.211	.712	.105		
Digit Span Backwards	.109	.228	.677	.101		
Openness	**.204**	**.016**	**4.68**	**.201**		
Music Training	.017	.854	.361	.016		
*Step 2*					.343	.281
Age	− .186	.058	1.78	− .161		
Gender	**.197**	**.009**	**7.33**	**.221**		
Education	.015	.882	.365	.013		
Nonverbal Reasoning	.106	.223	.700	.104		
Digit Span Forward	.045	.560	.420	.050		
Digit Span Backwards	.089	.290	.595	.090		
Openness	**.192**	**.015**	**4.95**	**.206**		
Music Training	− .183	.076	1.45	− .151		
Melody	.055	.593	.411	.046		
Pitch	** − .280**	**.009**	**7.62**	** − .222**		
Intensity	− .044	.616	.404	− .043		
Duration	− .116	.180	.803	− .114		
Gap Detection	− .139	.087	1.33	− .146		

Significant predictor variables are in bold font

## Discussion

Although links between musical expertise and speech processing are often attributed to music training (e.g., Patel, 2014; Slater et al., 2015), causal evidence is limited (Neves et al., 2022; Sala & Gobet, 2020; Schellenberg & Lima, 2024) and attempts to replicate frequently fail (Boebinger et al., 2015; Schellenberg et al., 2023; Whiteford et al., 2025). Recent work points instead to musical aptitude as the source of observed associations (Correia et al., 2022a, 2022b; Mankel & Bidelman, 2018; Swaminathan & Schellenberg, 2017). But what mechanisms link musical aptitude to speech processing? Our results answer this question by showing that (1) musical ability—particularly melody perception—predicts emotion recognition in speech prosody independently of music training, and (2) this association is explained by basic, domain-general pitch discrimination.

Bayesian analyses indicated that the apparent association between music training and prosodic emotion recognition was weak and disappeared after controlling for sociodemographic, cognitive, and personality covariates. This pattern suggests that associations reported in previous studies may reflect selection effects or confounding factors rather than a causal role of training. Although some older studies reported training advantages that survived control for cognitive ability (Lima & Castro, 2011; Thompson et al., 2004), more recent research with children (Neves et al., 2025) and adults (Correia et al., 2022a, 2022b) failed to find effects after adjusting for relevant covariates. Training could, in principle, enhance prosody recognition indirectly via cognitive improvements, which would explain why associations disappear when cognitive abilities are controlled. In a recent randomized trial, however, 2 years of music lessons did not improve prosodic emotion recognition (Neves et al., 2025), and causal evidence that music training enhances cognitive abilities is weak or nonexistent (Bigand & Tillmann, 2022; Sala & Gobet, 2020; Schellenberg & Lima, 2024).

By contrast, objective musical ability, most clearly melody discrimination, remained a reliable predictor of prosodic emotion recognition after accounting for training and control variables. Crucially, this association was specific to the auditory domain. Melody perception predicted prosodic but not facial emotion recognition, pointing to an auditory-specific mechanism (Correia et al., 2022a, 2022b). Cross-modal effects may still occur in developing or atypical populations, however, a hypothesis that future research could examine further. In children, for example, a correlation can emerge between musical ability and emotion recognition from faces, even though the link is stronger for prosody (Neves et al., 2025). Individuals with congenital amusia can also show deficits in both prosody and facial emotion processing (Lima et al., 2016; but see Zhishuai et al., 2017).

Our finding that melody, rather than rhythm, predicted prosodic emotion recognition replicates and extends prior evidence. In two studies, the melody subtest from the PROMS correlated with emotion recognition for pseudowords that conveyed emotions through multiple prosodic cues or pitch alone (Nussbaum et al., 2024, 2025). Reported associations with the rhythm subtest either disappeared when music training was controlled (Nussbaum et al., 2024) or were based on samples of trained participants (Nussbaum et al., 2025), which limits generalizability. In another study, children were tested with the Montreal Battery of Evaluation of Musical Abilities (Peretz et al., 2013) and the prosodic stimuli used here. Melody but not the rhythm subtest predicted prosodic emotion recognition (Neves et al., 2025). Altogether, these findings support the colloquial notion of prosody as the *melody of speech* and suggest that sensitivity to pitch changes over time is the principal contributor to the music–prosody overlap.

A key question concerns the processing level at which melodic and prosodic perception converge. The independence of the link from cognitive abilities, including short-term and working memory, argues against an explanation based on general memory for auditory sequences. Rather, our results point to low-level auditory sensitivity as the shared mechanism. Psychoacoustic thresholds correlated with both musical ability and prosodic emotion recognition. Consistent with Baldé et al. (2025) and models that place basic auditory processing at the foundation of musical abilities (Koelsch, 2011; Peretz & Coltheart, 2003), melody perception related strongly with pitch-related thresholds, whereas rhythm perception correlated with temporal and nontemporal thresholds, including pitch discrimination.

Although pitch discrimination emerged as the strongest predictor of prosodic emotion recognition, we also observed reliable associations with duration, intensity, and gap-detection thresholds, a finding in line with the notion that prosodic emotions are conveyed by multiple acoustic cues (Juslin & Laukka, 2003; Larrouy-Maestri et al., 2024). The prominent role of pitch is nevertheless consistent with results from Globerson et al. (2013), who found that two-tone pitch discrimination explained over 20% of the variance in prosodic emotion recognition. Similar findings have been observed in individuals with autism spectrum disorders (Globerson et al., 2015), and help to explain why emotion recognition remains high when prosody is conveyed solely by pitch cues (Nussbaum et al., 2024, 2025).

Importantly, when pitch thresholds and melody perception were considered simultaneously, only pitch discrimination contributed uniquely to explaining variance in a model that predicts prosodic emotion recognition. Higher-order melodic processing and other psychoacoustic predictors (duration, intensity, and gap-detection) failed to add explanatory power. Thus, even though multiple acoustic cues convey prosodic emotions, pitch discrimination appears to be the core building block shared by melody and prosody perception, which implies that the music–prosody link is primarily acoustic rather than music-specific. This interpretation is consistent with the auditory precision hypothesis (Saito, 2023; Saito & Tierney, 2025), which proposes that precise encoding of basic acoustic features underpins complex speech processing, including second-language learning.

But not all evidence aligns with this account. For example, Kempe et al. (2015) observed that musical ability predicted nonnative speech perception beyond pitch sensitivity (Kempe et al., 2015), and Nussbaum et al., (2024, 2025) reported that prosodic emotion recognition was more strongly correlated with melody than with a pitch test. Comparisons with Nussbaum et al.’s studies are difficult, however, because of methodological differences including smaller samples, a non-adaptive pitch task, and no direct comparison between melody and pitch. Future research could examine whether different facets of speech perception are associated differentially with basic auditory thresholds and higher-order musical abilities, and test further whether results are contingent on methodological choices.

Neurophysiological methods such as EEG could also clarify which stages of pitch perception best explain recognizing emotions from prosody. Adaptive pitch-discrimination tasks reflect both subcortical auditory precision (Marmel et al., 2013) and the efficiency of cortical processing at early automatic and later attentional stages (Tervaniemi et al., 2005). Individuals with better pitch thresholds may recognize prosodic emotions more accurately because of more precise early encoding or, alternatively, because of more efficient use of pitch cues in attentional-evaluative processes. Some EEG studies suggest that musician differences in prosody perception emerge at later stages (> 500 ms; Lehnen et al., 2025; Nussbaum et al., 2023), whereas others implicate subcortical (Strait et al., 2009) or early cortical mechanisms (Pinheiro et al., 2015). To our knowledge, no EEG study has yet tested whether individual differences in pitch sensitivity predict prosodic emotion recognition.

As with any correlational design, causal direction remains unresolved. Although it is theoretically grounded to hypothesize that pitch sensitivity supports both musical and prosodic processing, extensive experience with speech could also enhance pitch perception over time, indirectly improving melody perception. In any event, our findings point to a clear hypothesis for future testing: basic pitch sensitivity explains links between musical expertise and prosodic emotion recognition. More generally, our results suggest that a low-level auditory mechanism is the primary locus of overlap between musical expertise and emotional prosody. Our findings also reinforce the view that music training rarely improves nonmusical abilities (Neves et al., 2025; Schellenberg & Lima, 2024).

## Supplementary Information

Below is the link to the electronic supplementary material.

Supplementary file1 (DOCX 78.5 KB)

## Data Availability

The dataset generated and analyzed during the current study is available in the Open Science Framework (OSF) repository (https://osf.io/j692q/overview?view_only=fa4f6c681a444410b15b4f66752d49db).

## Footnotes

1 For completeness, associations between individual Gold-MSI subscales and the other primary measures (emotion recognition, psychoacoustic thresholds, objective musical abilities) are reported in the Supplementary Materials.
