# Supplementary material for: Musical ability and emotion recognition in speech prosody: The role of pitch discrimination
Source: Psychon Bull Rev. 2026 Feb 27;33(3):103. doi: 10.3758/s13423-026-02865-z (PMC12948796; doi:10.3758/s13423-026-02865-z)
Supplement: Supplementary file 1 — Supplementary file1 (DOCX 78.5 KB) [file 13423_2026_2865_MOESM1_ESM.docx]

**Supplementary Materials**

**Musical Ability and Emotion Recognition in Speech Prosody: The Role of Pitch Discrimination**

Aíssa M. Baldé^1^, E. Glenn Schellenberg^1,2^, & César F. Lima^1^

^1^Centro de Investigação e Intervenção Social (CIS-IUL),

Instituto Universitário de Lisboa (ISCTE-IUL)

^2^Department of Psychology, University of Toronto Mississauga

**Author Note**

Aíssa M. Baldé
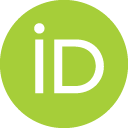
https://orcid.org/0000-0002-6161-516X

E. Glenn Schellenberg
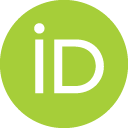
https://orcid.org/0000-0003-3681-6020

César F. Lima
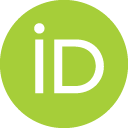
https://orcid.org/0000-0003-3058-7204

This work was funded by the Portuguese Foundation for Science and Technology (FCT) through a PhD studentship awarded to A.B. (SFRH/BD/2022.12352) and a Scientific Employment Stimulus grant awarded to E.G.S. (CEECIND/03266/2018).

E. Glenn Schellenberg and César F. Lima contributed equally to this work. Correspondence concerning this article should be addressed César F. Lima ([cesar.lima@iscte-iul.pt](mailto:cesar.lima@iscte-iul.pt)), Instituto Universitário de Lisboa (ISCTE-IUL), Av.ª das Forças Armadas, 1649-026 Lisboa, Portugal.

**Reanalysis of Correia, Castro, et al. (2022)**

Correia, Castro, et al. (2022, Table 3) found decisive evidence for a zero-order correlation between recognizing emotions from prosody and an aggregate measure of music-perception ability, *r* = -.395, *p* < .001, BF_10_ > 100. Their aggregate measure was formed by extracting the principal component from tests of pitch discrimination, duration discrimination, and musical beat perception. In our reanalysis, we considered correlations between prosody scores and each of the three original variables, with music training held constant. There was decisive evidence that prosody was correlated with thresholds for pitch discrimination, *r* = -.422, *p* < .001, BF_10_ > 100, and duration discrimination, *r* = -.288, *p* < .001, BF_10_ > 100, but no evidence for an association with beat perception, *r* = .135, *p* = .095, BF_10_ = .850. We also used multiple regression to model prosody scores as a function of pitch discrimination, duration discrimination, beat perception, and music training. The model explained 21.8% of the variance in prosody scores, *F*(4, 148) = 10.32, *p* < .001, BF_10_ > 100. Although pitch discrimination made a significant independent contribution to the model, *r* = -.346, *p* < .001, BF_10_ > 100, neither duration discrimination, *r* = -.135, *p* = .100, BF_10_ = .860, nor beat perception, *r* = -.067, *p* = .417, BF_10_ = .328, did so. Moreover, there was substantial evidence for a null partial association with beat perception.

**Supplementary Tables**

Tables S1 to S3 provide simple correlations between potential confounding variables and the primary measures: emotion recognition (Table S1), musical expertise (Table S2), and psychoacoustic thresholds (Table S3). Table S4 shows partial correlations between individual emotions in the prosody task and musical expertise measures. Control variables (age, gender, education, Digit Span Forward, Digit Span Backwards, nonverbal reasoning, and openness) were held constant. Table S5 reports partial correlations between psychoacoustic thresholds and the measures of emotion recognition and musical expertise, with music training held constant in addition to the control variables. Additionally, partial correlations between primary measures and subscales of the Gold-MSI are provided in Tables S6 (emotion recognition), S7 (musical expertise), and S8 (psychoacoustic thresholds).

**Table S1.**

*Associations Between Emotion Recognition and Potential Confounding Variables. Bold Font Indicates Significant Associations.*

|  |  |  | Prosody | Faces |
| --- | --- | --- | --- | --- |
| **Demographics** | Age | *r* | -.123 | -.046 |
|  |  | *p* | .117 | .162 |
|  |  | BF_10_ | .329 | .116 |
|  |  |  |  |  |
|  | Gender | *r* | .133 | **.294** |
|  |  | *p* | .090 | **<.001** |
|  |  | BF_10_ | .405 | **>100** |
|  |  |  |  |  |
|  | Education | *r* | .064 | .074 |
|  |  | *p* | .417 | .349 |
|  |  | BF_10_ | .135 | .151 |
|  |  |  |  |  |
| **Cognitive Abilities** | Nonverbal Reasoning | *r* | **.296** | .121 |
|  |  | *p* | **<.001** | .124 |
|  |  | BF_10_ | **>100** | .316 |
|  |  |  |  |  |
|  | Digit Span Forward | *r* | .176 | .000 |
|  |  | *p* | .024 | .995 |
|  |  | BF_10_ | 1.22 | .098 |
|  |  |  |  |  |
|  | Digit Span Backwards | *r* | **.243** | .098 |
|  |  | *p* | **.002** | .210 |
|  |  | BF_10_ | **12.8** | .213 |
|  |  |  |  |  |
| **Personality** | Extraversion | *r* | -.093 | -.037 |
|  |  | *p* | .236 | .639 |
|  |  | BF_10_ | .196 | .109 |
|  |  |  |  |  |
|  | Agreeableness | *r* | -.073 | .100 |
|  |  | *p* | .354 | .204 |
|  |  | BF_10_ | .150 | .217 |
|  |  |  |  |  |
|  | Conscientiousness | *r* | .021 | .003 |
|  |  | *p* | .791 | .969 |
|  |  | BF_10_ | .101 | .098 |
|  |  |  |  |  |
|  | Neuroticism | *r* | .055 | .089 |
|  |  | *p* | .484 | .259 |
|  |  | BF_10_ | .125 | .184 |
|  |  |  |  |  |
|  | Openness | *r* | .174 | .138 |
|  |  | *p* | .026 | .079 |
|  |  | BF_10_ | 1.13 | .453 |

**Table S2.**

*Associations Between Musical Expertise and Potential Confounding Variables. Bold Font Indicates Significant Associations.*

|  |  | Music  Training | Melody | Rhythm | Self-Reported  Musicality |
| --- | --- | --- | --- | --- | --- |
| Age | *r* | .195 | .168 | **.220** | .131 |
|  | *p* | .012 | .032 | **.005** | .094 |
|  | BF_10_ | 2.19 | .961 | **5.13** | .392 |
|  |  |  |  |  |  |
| Gender | *r* | -.113 | -.042 | .016 | -.087 |
|  | *p* | .151 | .598 | .835 | .267 |
|  | BF_10_ | .271 | .112 | .100 | .180 |
|  |  |  |  |  |  |
| Education | *r* | **.399** | **.277** | **.289** | **.218** |
|  | *p* | **<.001** | **<.001** | **<.001** | **.005** |
|  | BF_10_ | **>100** | **55.1** | **>100** | **4.83** |
|  |  |  |  |  |  |
| Nonverbal Reasoning | *r* | **.443** | **.391** | **.364** | **.283** |
|  | *p* | **<.001** | **<.001** | **<.001** | **<.001** |
|  | BF_10_ | **>100** | **>100** | **>100** | **79.3** |
|  |  |  |  |  |  |
| Digit Span Forward | *r* | **.231** | **.334** | **.392** | **.197** |
|  | *p* | **.003** | **<.001** | **<.001** | **.011** |
|  | BF_10_ | **8.01** | **>100** | **>100** | **2.34** |
|  |  |  |  |  |  |
| Digit Span Backwards | *r* | **.251** | **.320** | **.325** | .101 |
|  | *p* | **.001** | **<.001** | **<.001** | .199 |
|  | BF_10_ | **17.5** | **>100** | **>100** | .221 |
|  |  |  |  |  |  |
| Extraversion | *r* | .009 | .016 | -.075 | .085 |
|  | *p* | .910 | .839 | .339 | .282 |
|  | BF_10_ | .098 | .100 | .154 | .174 |
|  |  |  |  |  |  |
| Agreeableness | *r* | .146 | .150 | .079 | .141 |
|  | *p* | .063 | .057 | .312 | .072 |
|  | BF_10_ | .545 | .592 | .162 | .484 |
|  |  |  |  |  |  |
| Conscientiousness | *r* | .005 | .027 | -.030 | .091 |
|  | *p* | .948 | .733 | .703 | .246 |
|  | BF_10_ | .098 | .104 | .105 | .191 |
|  |  |  |  |  |  |
| Neuroticism | *r* | -.015 | -.038 | -.027 | -.071 |
|  | *p* | .848 | .634 | .728 | .369 |
|  | BF_10_ | .100 | .110 | .104 | .146 |
|  |  |  |  |  |  |
| Openness | *r* | **.377** | **.261** | **.236** | **.382** |
|  | *p* | **<.001** | **<.001** | **.002** | **<.001** |
|  | BF_10_ | **>100** | **27.0** | **9.53** | **>100** |

**Table S3.**

*Associations Between Psychoacoustic Thresholds and Potential Confounding Variables.*

|  |  | | Pitch | | Intensity | | Duration | | Complex  Duration | | Contour Speed | | Backward  Masking | | Gap  Detection | | Timbre | | Scale  Mistuning | |  |
| --- | --- | --- | --- | --- | --- | --- | --- | --- | --- | --- | --- | --- | --- | --- | --- | --- | --- | --- | --- | --- | --- |
| Age | | *r* | | -.099 | | -.145 | | -.177 | | **-.208** | | -.001 | | -.144 | | .171 | | .205 | | -.189 | |
|  | | *p* | | .215 | | .066 | | .025 | | **.008** | | .989 | | .066 | | .030 | | .010 | | .017 | |
|  | | BF_10_ | | .212 | | .522 | | 1.20 | | **3.20** | | .101 | | .521 | | 1.01 | | 2.63 | | 1.66 | |
|  | |  | |  | |  | |  | |  | |  | |  | |  | |  | |  | |
| Gender | | *r* | | .135 | | **.254** | | .052 | | .057 | | .074 | | .104 | | .010 | | .092 | | .127 | |
|  | | *p* | | .090 | | **.001** | | .509 | | .469 | | .359 | | .187 | | .903 | | .254 | | .111 | |
|  | | BF_10_ | | .412 | | **19.1** | | .122 | | .127 | | .153 | | .232 | | .100 | | .191 | | .350 | |
|  | |  | |  | |  | |  | |  | |  | |  | |  | |  | |  | |
| Education | | *r* | | **-.224** | | **-.215** | | **-.212** | | **-.234** | | -.050 | | -.166 | | -.053 | | .157 | | **-.280** | |
|  | | *p* | | **.004** | | **.006** | | **.007** | | **.003** | | .537 | | .034 | | .510 | | .050 | | **<.001** | |
|  | | BF_10_ | | **5.49** | | **4.12** | | **3.67** | | **8.50** | | .121 | | .913 | | .123 | | .668 | | **56.8** | |
|  | |  | |  | |  | |  | |  | |  | |  | |  | |  | |  | |
| Nonverbal Reasoning | | *r* | | **-.387** | | -.177 | | **-.295** | | -.181 | | **-.351** | | **-.364** | | **-.275** | | -.153 | | **-.345** | |
|  | | *p* | | **<.001** | | .024 | | **<.001** | | .021 | | **<.001** | | **<.001** | | **<.001** | | .056 | | **<.001** | |
|  | | BF_10_ | | **>100** | | 1.22 | | **>100** | | 1.36 | | **>100** | | **>100** | | **45.3** | | .611 | | **>100** | |
|  | |  | |  | |  | |  | |  | |  | |  | |  | |  | |  | |
| Digit Span Forward | | *r* | | **-.232** | | -.110 | | -.188 | | -.147 | | **-.260** | | -.175 | | -.203 | | -.109 | | **-.210** | |
|  | | *p* | | **.003** | | .162 | | .017 | | .062 | | **.001** | | .025 | | .010 | | .175 | | **.008** | |
|  | | BF_10_ | | **7.42** | | .259 | | 1.66 | | .553 | | **20.1** | | 1.18 | | 2.67 | | .250 | | **3.23** | |
|  | |  | |  | |  | |  | |  | |  | |  | |  | |  | |  | |
| Digit Span Backwards | | *r* | | **-.237** | | -.178 | | -.196 | | **-.217** | | **-.227** | | **-.222** | | -.207 | | -.112 | | -.175 | |
|  | | *p* | | **.003** | | .024 | | .013 | | **.006** | | **.004** | | **.004** | | .009 | | .164 | | .027 | |
|  | | BF_10_ | | **9.14** | | 1.25 | | 2.11 | | **4.41** | | **5.46** | | **5.67** | | 2.99 | | .262 | | 1.11 | |
|  | |  | |  | |  | |  | |  | |  | |  | |  | |  | |  | |
| Extraversion | | *r* | | .036 | | -.035 | | .033 | | .058 | | .046 | | -.040 | | .163 | | -.009 | | .029 | |
|  | | *p* | | .651 | | .658 | | .678 | | .467 | | .572 | | .610 | | .040 | | .913 | | .713 | |
|  | | BF_10_ | | .109 | | .108 | | .107 | | .128 | | .118 | | .111 | | .805 | | .101 | | .106 | |
|  | |  | |  | |  | |  | |  | |  | |  | |  | |  | |  | |

|  |  |  |  |  |  |  |  |  |  |  |
| --- | --- | --- | --- | --- | --- | --- | --- | --- | --- | --- |
|  |  | Pitch | Intensity | Duration | Complex  Duration | Contour Speed | Backward  Masking | Gap  Detection | Timbre | Scale  Mistuning |
| Agreeableness | *r* | -.111 | -.102 | -.082 | .017 | .022 | -.112 | .115 | .065 | -.124 |
|  | *p* | .162 | .198 | .304 | .831 | .784 | .152 | .149 | .422 | .119 |
|  | BF_10_ | .261 | .223 | .166 | .101 | .104 | .271 | .277 | .138 | .331 |
|  |  |  |  |  |  |  |  |  |  |  |
| Conscientiousness | *r* | -.061 | .010 | -.089 | .116 | .041 | -.059 | -.047 | -.104 | .023 |
|  | *p* | .444 | .898 | .264 | .141 | .615 | .450 | .555 | .197 | .776 |
|  | BF_10_ | .132 | .099 | .183 | .287 | .114 | .130 | .118 | .228 | .103 |
|  |  |  |  |  |  |  |  |  |  |  |
| Neuroticism | *r* | .028 | .079 | .107 | -.130 | -.048 | -.015 | -.003 | .020 | .044 |
|  | *p* | .727 | .316 | .177 | .099 | .555 | .847 | .968 | .801 | .584 |
|  | BF_10_ | .105 | .162 | .243 | .378 | .119 | .100 | .099 | .103 | .115 |
|  |  |  |  |  |  |  |  |  |  |  |
| Openness | *r* | **-.236** | -.072 | -.156 | **-.211** | -.110 | -.140 | -.061 | .039 | **-.341** |
|  | *p* | **.003** | .360 | .048 | **.007** | .173 | .074 | .447 | .629 | **<.001** |
|  | BF_10_ | **8.59** | .149 | .682 | **3.61** | .252 | .473 | .132 | .112 | **>100** |

**Table S4.**

*Partial Pairwise Associations Between Musical Expertise and Recognizing Emotions in Prosody, Reported Separately for Each Emotion. Age, Gender, Education, Nonverbal Reasoning, Digit Span Forward, Digit Span Backwards, and Openness were Held Constant.*

| Emotion |  | Music  Training | Melody | Rhythm | Self-Reported  Musicality |
| --- | --- | --- | --- | --- | --- |
| Happiness | *r* | -.024 | -.001 | -.090 | .030 |
|  | *p* | .769 | .992 | .262 | .713 |
|  | BF_10_ | .428 | .414 | .698 | .437 |
|  |  |  |  |  |  |
| Fear | *r* | -.034 | .132 | .100 | .016 |
|  | *p* | .668 | .102 | .214 | .842 |
|  | BF_10_ | .409 | 1.20 | .734 | .385 |
|  |  |  |  |  |  |
| Neutral | *r* | .083 | **.203** | .118 | **.222** |
|  | *p* | .303 | **.011** | .140 | **.005** |
|  | BF_10_ | .616 | **6.14** | .992 | **11.3** |
|  |  |  |  |  |  |
| Disgust | *r* | .069 | .128 | .090 | .056 |
|  | *p* | .388 | .112 | .263 | .485 |
|  | BF_10_ | .552 | 1.17 | .682 | .494 |
|  |  |  |  |  |  |
| Anger | *r* | -.088 | -.030 | -.001 | .052 |
|  | *p* | .276 | .707 | .991 | .517 |
|  | BF_10_ | .600 | .382 | .357 | .429 |
|  |  |  |  |  |  |
| Surprise | *r* | .124 | **.190** | .160 | .150 |
|  | *p* | .123 | **.017** | .046 | .061 |
|  | BF_10_ | 1.01 | **4.30** | 2.05 | 1.66 |
|  |  |  |  |  |  |
| Sadness | *r* | -.045 | **.207** | .097 | .064 |
|  | *p* | .579 | **.009** | .228 | .427 |
|  | BF_10_ | .479 | **7.07** | .772 | .548 |
|  |  |  |  |  |  |

**Table S5.**

*Partial Associations Between Psychoacoustic Thresholds and Measures of Emotion Recognition and Musical Expertise. Age, Gender, Education, Nonverbal Reasoning, Digit Span Forward, Digit Span Backwards, Openness, and Music Training Were Held Constant. Bold Font Indicates Significant Associations.*

|  |  | Prosody | Faces | Melody | Rhythm | Self-Reported  Musicality |
| --- | --- | --- | --- | --- | --- | --- |
| Pitch | *r* | **-.370** | **-.200** | **-.439** | **-.199** | **-.249** |
|  | *p* | **<.001** | **.013** | **<.001** | **.014** | **.002** |
|  | BF_10_ | **>100** | **5.36** | **>100** | **4.52** | **21.6** |
|  |  |  |  |  |  |  |
| Intensity | *r* | **-.226** | **-.209** | **-.231** | -.169 | **-.274** |
|  | *p* | **.005** | **.009** | **.004** | .037 | **<.001** |
|  | BF_10_ | **11.8** | **7.17** | **11.6** | 2.04 | **62.5** |
|  |  |  |  |  |  |  |
| Duration | *r* | **-.279** | **-.183** | **-.226** | **-.257** | -.089 |
|  | *p* | **<.001** | **.023** | **.005** | **.001** | .276 |
|  | BF_10_ | **76.2** | **3.54** | **9.51** | **31.7** | .411 |
|  |  |  |  |  |  |  |
| Complex Duration | *r* | -.158 | -.068 | **-.296** | **-.217** | -.122 |
|  | *p* | .051 | .400 | **<.001** | **.007** | .132 |
|  | BF_10_ | 1.91 | .534 | **>100** | **7.90** | .686 |
|  |  |  |  |  |  |  |
| Contour Speed | *r* | -.068 | -.101 | **-.374** | -.184 | -.118 |
|  | *p* | .413 | .223 | **<.001** | .026 | .153 |
|  | BF_10_ | .514 | .757 | **>100** | 2.82 | .627 |
|  |  |  |  |  |  |  |
| Backward Masking | *r* | -.145 | -.117 | **-.301** | **-.229** | **-.205** |
|  | *p* | .070 | .147 | **<.001** | **.004** | **.010** |
|  | BF_10_ | 1.52 | .964 | **>100** | **12.4** | **5.17** |
|  |  |  |  |  |  |  |
| Gap Detection | *r* | **-.239** | .013 | **-.250** | -.007 | **-.226** |
|  | *p* | **.003** | .875 | **.002** | .929 | **.005** |
|  | BF_10_ | **17.4** | .402 | **21.8** | .270 | **9.37** |
|  |  |  |  |  |  |  |
| Timbre | *r* | -.146 | -.069 | **-.331** | -.128 | **-.239** |
|  | *p* | 077 | .403 | **<.001** | .121 | **.003** |
|  | BF_10_ | 1.46 | .548 | **>100** | .843 | **13.6** |
|  |  |  |  |  |  |  |
| Scale Mistuning | *r* | -.138 | .040 | **-.369** | -.124 | **-.259** |
|  | *p* | .091 | .628 | **<.001** | .128 | **.001** |
|  | BF_10_ | 1.27 | .431 | **>100** | .801 | **31.2** |

**Table S6.** *Partial Associations Between Gold-MSI Subscales and Emotion Recognition. Age, Gender, Education, Nonverbal Reasoning, Digit Span Forward, Digit Span Backwards, and Openness Were Held Constant. Bold Font Indicates Significant Associations.*

| Gold-MSI  Subscale |  | Prosody | Faces |
| --- | --- | --- | --- |
|  |  |  |  |
| Active Engagement | *r* | -.074 | .034 |
|  | *p* | .354 | .671 |
|  | BF_10_ | .502 | .404 |
|  |  |  |  |
| Perceptual Abilities | *r* | .127 | -.008 |
|  | *p* | .112 | .919 |
|  | BF_10_ | .954 | .376 |
|  |  |  |  |
| Singing Abilities | *r* | .128 | -.005 |
|  | *p* | .112 | .954 |
|  | BF_10_ | .952 | .375 |
|  |  |  |  |
| Emotions | *r* | -.030 | .014 |
|  | *p* | .705 | .863 |
|  | BF_10_ | .366 | .379 |

*Note.* The Music Training subscale of the Gold-MSI was omitted because it constituted a primary variable that had already been examined elsewhere.

**Table S7.** *Partial Associations Between Gold-MSI Subscales and Scores on the MET. Age, Gender, Education, Nonverbal Reasoning, Digit Span Forward, Digit Span Backwards, and Openness Were Held Constant. Bold Font Indicates Significant Associations.*

| Gold-MSI  Subscale |  | Melody | Rhythm |
| --- | --- | --- | --- |
|  |  |  |  |
|  |  |  |  |
| Active Engagement | *r* | .024 | .034 |
|  | *p* | .766 | .676 |
|  | BF_10_ | .321 | .319 |
|  |  |  |  |
| Perceptual Abilities | *r* | **.385** | **.233** |
|  | *p* | **<.001** | **.003** |
|  | BF_10_ | **>100** | **15.2** |
|  |  |  |  |
|  |  |  |  |
| Singing Abilities | *r* | **.494** | **.226** |
|  | *p* | **<.001** | **.004** |
|  | BF_10_ | **>100** | **12.0** |
|  |  |  |  |
| Emotions | *r* | .150 | .106 |
|  | *p* | .062 | .188 |
|  | BF_10_ | 1.50 | .649 |

*Note.* The Music Training subscale of the Gold-MSI was omitted because it constituted a primary variable that had already been examined elsewhere.

**Table S8.** *Partial Associations Between Gold-MSI Subscales and Psychoacoustic Thresholds. Age, Gender, Education, Nonverbal Reasoning, Digit Span Forward, Digit Span Backwards, Openness, and Music Training Were Held Constant. Bold Font Indicates Significant Associations.*

|  |  | Gold-MSI Subscale | | | |
| --- | --- | --- | --- | --- | --- |
|  |  | Active Engagement | Perceptual  Abilities | Singing  Abilities | Emotions |
| Pitch | *r* | -.017 | **-.405** | **-.389** | -.038 |
|  | *p* | .833 | **<.001** | **<.001** | .637 |
|  | BF_10_ | .346 | **>100** | **>100** | .375 |
|  |  |  |  |  |  |
| Intensity | *r* | -.019 | **-.294** | **-.215** | -.054 |
|  | *p* | .818 | **<.001** | **.007** | .503 |
|  | BF_10_ | .401 | **>100** | **8.71** | .474 |
|  |  |  |  |  |  |
| Duration | *r* | .005 | **-.187** | -.050 | .064 |
|  | *p* | .955 | **.020** | .539 | .429 |
|  | BF_10_ | .388 | **3.94** | .455 | .506 |
|  |  |  |  |  |  |
| Complex Duration | *r* | .061 | -.106 | -.117 | -.064 |
|  | *p* | .451 | .190 | .147 | .431 |
|  | BF_10_ | .509 | .827 | .974 | .520 |
|  |  |  |  |  |  |
| Contour Speed | *r* | -.134 | **-.299** | **-.198** | .007 |
|  | *p* | .103 | **<.001** | **.016** | .936 |
|  | BF_10_ | 1.16 | **>100** | **4.57** | .368 |
|  |  |  |  |  |  |
| Backward Masking | *r* | .045 | **-.320** | **-.291** | -.040 |
|  | *p* | .579 | **<.001** | **<.001** | .619 |
|  | BF_10_ | .419 | **>100** | **>100** | .408 |
|  |  |  |  |  |  |
| Gap Detection | *r* | .072 | **-.234** | **-.210** | .112 |
|  | *p* | .377 | **.004** | **.009** | .169 |
|  | BF_10_ | .512 | **14.8** | **7.12** | .832 |
|  |  |  |  |  |  |
| Timbre | *r* | .063 | **-.210** | **-.217** | -.052 |
|  | *p* | .446 | **.010** | **.008** | .530 |
|  | BF_10_ | .517 | **6.62** | **8.18** | .478 |
|  |  |  |  |  |  |
| Scale Mistuning | *r* | -.014 | **-.330** | **-.350** | -.178 |
|  | *p* | .863 | **<.001** | **<.001** | .028 |
|  | BF_10_ | .338 | **>100** | **>100** | 2.86 |

*Note.* The Music Training subscale of the Gold-MSI was omitted because it constituted a primary variable that had already been examined elsewhere.
